# Supplementary material for: Clinical manifestations, prevalence, risk factors, outcomes, transmission, diagnosis and treatment of COVID-19 in pregnancy and postpartum: a living systematic review protocol
Source: BMJ Open. 2020 Dec 2;10(12):e041868. doi: 10.1136/bmjopen-2020-041868 (PMC7712931; doi:10.1136/bmjopen-2020-041868)
Supplement: Supplementary data [file bmjopen-2020-041868supp001.pdf]

Appendix 1: Pubmed search strategy to be used in the living systematic review on COVID-19 in pregnant and recently pregnant women

Item	Term
1	pregnancy/
2	pregnan*.tw.
3	neonatal.tw.
4	perinatal.tw.
5	mothers/.
6	mother.tw.
7	maternal.tw.
8	obstetric.tw.
9	infant, newborn/
10	infant.tw.
11	newborn.tw.
12	child*.tw.
13	or/1-12
14	COVID-19.tw.
15	COVID-2019.tw.
16	severe acute respiratory syndrome coronavirus 2.tw.
17	2019-nCoV.tw.
18	SARS-CoV-2.tw.
19	2019nCoV.tw
20	or/14-19
21	coronavirus.tw.
22	2019/12.pd
23	2020.pd.
24	or/22-23
25	21 and 24
24	or/20-25
25	13 and 24
