# Supplementary material for: Clinical manifestations, prevalence, risk factors, outcomes, transmission, diagnosis and treatment of COVID-19 in pregnancy and postpartum: a living systematic review protocol
Source: BMJ Open. 2020 Dec 2;10(12):e041868. doi: 10.1136/bmjopen-2020-041868 (PMC7712931; doi:10.1136/bmjopen-2020-041868)
Supplement: Author's manuscript [file bmjopen-2020-041868.draft_revisions.pdf]

BMJ Open is committed to open peer review. As part of this commitment we make the peer review history of every article we publish publicly available.

When an article is published we post the peer reviewers' comments and the authors' responses online. We also post the versions of the paper that were used during peer review. These are the versions that the peer review comments apply to.

The versions of the paper that follow are the versions that were submitted during the peer review process. They are not the versions of record or the final published versions. They should not be cited or distributed as the published version of this manuscript.

BMJ Open is an open access journal and the full, final, typeset and author-corrected version of record of the manuscript is available on our site with no access controls, subscription charges or pay-per-view fees (http://bmjopen.bmj.com).

If you have any questions on BMJ Open's open peer review process please email info.bmjopen@bmj.com

BMJ Open

Clinical manifestations, prevalence, risk factors, outcomes, transmission, diagnosis and treatment of coronavirus disease (COVID-19) in pregnancy and postpartum: A living systematic review protocol

Journal:	*BMJ Open*
Manuscript ID	bmjopen-2020-041868
Article Type:	Protocol
Date Submitted by the Author:	19-Jun-2020
Complete List of Authors:	Yap, Magnus; University of Birmingham College of Medical and Dental Sciences<br>Debenham, Luke; University of Birmingham College of Medical and Dental Sciences<br>Kew, Tania; University of Birmingham College of Medical and Dental Sciences<br>Chatterjee, Shaunak; University of Birmingham College of Medical and Dental Sciences<br>Allotey, John; University of Birmingham Institute of Applied Health Research; University of Birmingham, WHO Collaborating Centre for Global Women's Health, Institute of Metabolism and Systems Research<br>Stallings, Elena; IRYCIS, Clinical Biostatistics Unit; CIBERESP<br>Coomar, Dyuti; University of Birmingham Institute of Applied Health Research<br>Lee, Siang; University of Birmingham Institute of Applied Health Research<br>Qiu, xiu; Guangzhou Women and Children's Medical Center, Division of Birth Cohort Study; Guangzhou Women and Children's Medical Center, Department of Obstetrics and Gynecology<br>Yuan, Mingyang; Guangzhou Women and Children's Medical Center, Division of Birth Cohort Study<br>Clavé Llavall, Anna; University of Birmingham College of Medical and Dental Sciences<br>Dixit, Anushka; University of Birmingham College of Medical and Dental Sciences<br>Zhou, Dengyi; University of Birmingham College of Medical and Dental Sciences<br>Balaji, Rishab; University of Birmingham College of Medical and Dental Sciences<br>van Wely, Madelon; Amsterdam University Medical Centres, Netherlands Satellite of the Cochrane Gynaecology and Fertility Group<br>Kostova, Elena; Amsterdam University Medical Centres, Netherlands Satellite of the Cochrane Gynaecology and Fertility Group<br>van Leeuwen, Elisabeth; Amsterdam University Medical Centres, Department of Obstetrics and Gynaecology<br>Mofenson, Lynne; Elizabeth Glaser Pediatric AIDS Foundation,<br>Kunst, Heinke; Queen Mary University of London, Department of Respiratory Medicine

Khalil, Asma; St George's University of London
Tiberi, Simon; Barts Health NHS Trust, Blizard Institute, Queen Mary University of London, Department of Infection; Barts Health NHS Trust, Department of Infectious Diseases
Thomas, James; University College London Social Science Research Unit
Brizuela, Vanessa; WHO, Department of Reproductive Health and Research
Broutet, Nathalie; WHO, Department of Reproductive Health and Research
Kara, Edna; WHO, Department of Sexual and Reproductive Health and Research
Kim, Caron; WHO, Department of Sexual and Reproductive Health and Research
Thorson, Anna; WHO, Department of Sexual and Reproductive Health and Research
Rayco-Solon, Pura; WHO, The Department of Maternal, Newborn, Child and Adolescent Health
Pardo-Hernandez, Hector; WHO, Cross-cutting Global Initiatives/Nutrition and Food Safety department
Oladapo, Olufemi ; WHO, Department of Sexual and Reproductive Health and Research
Zamora , Javier ; IRYCIS, Clinical Biostatistics Unit; CIBERESP
Bonet, Mercedes; WHO, Department of Sexual and Reproductive Health and Research
Thangaratinam, Shakila; Birmingham Women's and Children's Hospitals NHS Foundation Trust; University of Birmingham, WHO Collaborating Centre for Global Women's Health, Institute of Metabolism and Systems Research

Keywords: Maternal medicine < OBSTETRICS, VIROLOGY, NEONATOLOGY, Protocols & guidelines < HEALTH SERVICES ADMINISTRATION & MANAGEMENT, PERINATOLOGY

SCHOLARONE™
Manuscripts

I, the Submitting Author has the right to grant and does grant on behalf of all authors of the Work (as defined in the below author licence), an exclusive licence and/or a non-exclusive licence for contributions from authors who are: i) UK Crown employees; ii) where BMJ has agreed a CC-BY licence shall apply, and/or iii) in accordance with the terms applicable for US Federal Government officers or employees acting as part of their official duties; on a worldwide, perpetual, irrevocable, royalty-free basis to BMJ Publishing Group Ltd ("BMJ") its licensees and where the relevant Journal is co-owned by BMJ to the co-owners of the Journal, to publish the Work in this journal and any other BMJ products and to exploit all rights, as set out in our licence.

The Submitting Author accepts and understands that any supply made under these terms is made by BMJ to the Submitting Author unless you are acting as an employee on behalf of your employer or a postgraduate student of an affiliated institution which is paying any applicable article publishing charge ("APC") for Open Access articles. Where the Submitting Author wishes to make the Work available on an Open Access basis (and intends to pay the relevant APC), the terms of reuse of such Open Access shall be governed by a Creative Commons licence – details of these licences and which Creative Commons licence will apply to this Work are set out in our licence referred to above.

Other than as permitted in any relevant BMJ Author's Self Archiving Policies, I confirm this Work has not been accepted for publication elsewhere, is not being considered for publication elsewhere and does not duplicate material already published. I confirm all authors consent to publication of this Work and authorise the granting of this licence.

For peer review only

Clinical manifestations, prevalence, risk factors, outcomes, transmission, diagnosis and treatment of coronavirus disease (COVID-19) in pregnancy and postpartum: A living systematic review protocol

Magnus Yap*,[1] Luke Debenham*,[1] Tania Kew*,[1] Shaunak Chatterjee*,[1] John Allotey,[2,3] Elena Stallings,[4,5] Dyuti Coomar,[2] Siang Ing Lee,[2] Xiu Qui,[6,7,8] Mingyang Yuan,[2,6], Anna Clavé Llavall,[1] Anushka Dixit,[1] Dengyi Zhou,[1] Rishab Balaji,[1] Madelon van Wely,[9] Elena Kostova,[9] Elisabeth van Leeuwen,[10] Lynne Mofenson,[11] Heinke Kunst,[12] Asma Khalil,[13] Simon Tiberi,[12,14] James Thomas,[15] Vanessa Brizuela,[16] Nathalie Broutet,[16] Edna Kara,[16] Caron Rahn Kim,[16] Anna Thorson,[16] Paro Rayco-Solon,[16] Hector Pardo-Hernandez,[16] Olufemi Taiwo Oladapo,[16] Javier Zamora,[4,5,17] Mercedes Bonet,[16] Shakila Thangaratinam[3,18] for PregCOV-19 Consortium

[1]Birmingham Medical School, University of Birmingham, Birmingham, United Kingdom
[2]Institute of Applied Health Research, University of Birmingham, Birmingham, United Kingdom
[3]WHO Collaborating Centre for Global Women's Health, Institute of Metabolism and Systems Research, University of Birmingham, Birmingham, United Kingdom
[4]Clinical Biostatistics Unit, Hospital Universitario Ramón y Cajal (IRYCIS), Madrid, Spain
[5]CIBER Epidemiology and Public Health (CIBERESP), Madrid, Spain
[6]Division of Birth Cohort Study, Guangzhou Women and Children's Medical Center, Guangzhou Medical University, Guangzhou, China
[7]Department of Woman and Child Health Care, Guangzhou Women and Children's Medical Center, Guangzhou Medical University, Guangzhou, China
[8]Department of Obstetrics and Gynecology, Guangzhou Women and Children's Medical Center, Guangzhou Medical University, Guangzhou, China
[9]Netherlands Satellite of the Cochrane Gynaecology and Fertility Group, Amsterdam University Medical Center, Amsterdam, The Netherlands
[10]Department of Obstetrics and Gynaecology, Amsterdam University Medical Center, Amsterdam, The Netherlands
[11]Elizabeth Glaser Pediatric AIDS Foundation, Washington DC, United States
[12]Blizard Institute, Queen Mary University of London, London, United Kingdom
[13]St George's University London, London, United Kingdom
[14]Department of Infectious Diseases, Barts Health NHS Trust, London, United Kingdom
[15]UCL Institute of Education, University College London, London, United Kingdom
[16]UNDP/UNFPA/UNICEF/WHO/World Bank Special Programme of Research, Development and Research Training in Human Reproduction (HRP), Department of Sexual and Reproductive Health and Research, World Health Organization, Avenue Appia 20, 1211, Geneva, Switzerland
[17]Women's Health Research Unit, Queen Mary University of London, London, United Kingdom
[18]Birmingham Women's and Children's NHS Foundation Trust, Birmingham, United Kingdom

*Joint first authors

For peer review only

Corresponding author: John Allotey, Institute of Applied Health Research, University of Birmingham, College of Medical and Dental Sciences, Birmingham, United Kingdom.
Email: j.allotey.1@bham.ac.uk

Word count: 2765

Keywords: COVID-19, pregnancy, postpartum, neonate, living systematic review, meta-analysis, prevalence, risk factors, outcomes, vertical transmission, diagnosis, diagnostic tests, treatment,

For peer review only

Abstract

Introduction

Rapid, robust and continually updated evidence synthesis is required to inform management of COVID-19 (coronavirus disease) in pregnant and postpartum women, and keep pace with the emerging evidence during the pandemic.

Methods and analysis

We plan to undertake a living systematic review to assess the prevalence, clinical manifestations, risk factors, rates of maternal and perinatal complications, potential for mother-to-child transmission, accuracy of diagnostic tests, and effectiveness of treatment for COVID-19 in pregnant and postpartum women (including after miscarriage or abortion). We will search Medline, Embase, World Health Organization COVID-19 database, preprint servers, the China National Knowledge Infrastructure system and Wanfang databases from inception. We will supplement our search with studies mapped by Cochrane Fertility and Gynaecology group, EPPI-Centre, COVID-19 study repositories, reference lists, and social media blogs. The search will be updated every week and not be restricted by language.

We will include observational cohort (with ≥10 participants) and randomised studies reporting on prevalence of COVID-19 in pregnant and postpartum women, and the rates of clinical manifestations and outcomes, and risk factors in pregnant and postpartum women alone or in comparison with non-pregnant women with COVID-19 or pregnant women without COVID-19, and studies on tests and treatments for COVID-19. We will additionally include case reports and case series with evidence on mother-to-child transmission of SARS-CoV-2 in utero, intrapartum or postpartum.

For peer review only - http://bmjopen.bmj.com/site/about/guidelines.xhtml

We will appraise the quality of the included studies using appropriate tools to assess the risk of bias. At least two independent reviewers will undertake study selection, quality assessment and data extraction every two weeks. We will synthesise the findings using quantitative random effects meta-analysis and report odds ratios (OR) or proportions with 95% confidence intervals and prediction intervals. Case reports and series will be reported as qualitative narrative synthesis. Heterogeneity will be reported as I^2 and tau-squared statistics.

Word count – 300

Ethics and dissemination: Ethical approval is not required as this is a synthesis of primary data. Monthly updates of the results will be published on a dedicated website and disseminated through publications, social media and webinars.

Protocol registration number on PROSPERO: CRD42020178076

Summary - 'Strengths and limitations of this study'

- Our living systematic review will be underpinned by a comprehensive literature search, study quality assessment, and appropriate planned meta-analysis to efficiently collate the overall findings on COVID-19 in pregnant and postpartum women.
- We will continuously update our search, study selection, data extraction, analysis and reporting of the findings at pre-specified time intervals.
- Rapid publication of new studies with new outcomes of interest, screening and testing strategies and reporting of novel treatments may require changes in the review protocol, specifically regarding data extraction and search strategies for future updates.

For peer review only

- This review may be subject to publication bias, since studies with perceived positive results may be published faster than those with perceived negative results. We will continuously review registries of randomised controlled trials to detect trials that should have reported results but that have not done so and will contact corresponding authors to obtain results.
- The dynamic nature of the living systematic review requires dedicated team of committed researchers and resources, efficient peer review and support of the journal editors, to ensure that findings are rapidly published in the public domain with provision for continuous updates to inform living guidelines and policies.

For peer review only

For peer review only - http://bmjopen.bmj.com/site/about/guidelines.xhtml

INTRODUCTION

Coronavirus disease (COVID-19) caused by the severe acute respiratory syndrome coronavirus 2 (SARS-CoV-2) was declared a pandemic by the World Health Organization (WHO) on May 11, 2020.[1] In the first three months of the pandemic alone, over 4.5 million individuals have been affected - the infection continues to spread rapidly.[2] Case fatality rates range from 0.4-3.6% depending on the country and detection method.[3,4] In previous serious coronavirus outbreaks caused by SARS and MERS (Middle East Respiratory Syndrome), the rates of intensive care unit admission and mortality were significantly higher in infected pregnant than non-pregnant women, and adverse pregnancy outcomes were common.[5] There are concerns about the potential effects of SARS-CoV-2 infection on mothers and babies, including the risks of transmission to the foetus and neonate.[6] Some countries such as the UK have classed pregnant women as a vulnerable group requiring shielding during the pandemic.[7] Black and ethnic minority individuals are considered to be more likely to be infected and have severe COVID-19 disease than Caucasian individuals.[8-10]

There has been a rapid increase in the numbers of published studies and reports on the prevalence of SARS-CoV-2 infection in pregnancy, risk factors, mother-to-child transmission, effects on pregnant and recently pregnant women, including those who have delivered or had a recent abortion, and their babies.[11] Traditional systematic reviews are not able to keep pace with the rapid pace of publications, and quickly become outdated.[12] Furthermore, numerous systematic reviews, addressing similar questions and including identical numbers of studies that differ very slightly from each other, make it challenging for guideline makers to identify the up to-date evidence.[13,14] Many of these reviews do not follow reporting guidelines and other general principles of conducting robust systematic

For peer review only - http://bmjopen.bmj.com/site/about/guidelines.xhtml

reviews, often including case series and case control studies in the meta-analysis resulting in biased estimates.

Any recommendation on the care of pregnant women and recently pregnant women with suspected or confirmed COVID-19, and their babies, should be based on robust evidence. Clinicians need a single point of reference that comprehensively provides up-to-date evidence for key questions. In a rapidly changing research and clinical environment, this requires a clear prospective plan to update the available evidence beyond conventional systematic reviews and meta-analyses. We propose to undertake a living systematic review to address the key research questions on SARS-CoV-2 infection in pregnant and postpartum women, including after childbirth and early pregnancy loss.

AIM

Our goal is to provide up to-date evidence on the risks and risk factors for COVID-19 and associated complications in pregnant and postpartum women and their babies through a living systematic review and meta-analysis, and to assess the accuracy of diagnostic tests and effectiveness of treatment in the management of the disease.

For peer review only

OBJECTIVES

In pregnant and postpartum women (including after miscarriage or abortion):

- To determine the prevalence of and risk factors for SARS-CoV-2 infection and severe COVID-19.
- To evaluate the accuracy of tests and prediction models for screening, diagnosis and prediction of COVID-19 and its complications.
- To assess the effects of interventions for prevention of SARS-CoV-2 infection.

For peer review only - http://bmjopen.bmj.com/site/about/guidelines.xhtml

In pregnant and postpartum women (including after miscarriage) with suspected or confirmed COVID-19:

- To study of rates of clinical symptoms and signs, laboratory and radiological manifestations of the disease, and compare against non-pregnant women.
- To assess the rates and risk factors for COVID-related and pregnancy-related maternal and perinatal outcomes and compare against non-pregnant women with COVID-19, and pregnant women without the disease respectively.
- To determine the risks and risk factors for mother-child transmission of SARS-CoV-2 in utero, intra and peripartum, and the prevalence and persistence of the viral particles or immunological response in breast milk, amniotic fluid, cord blood, placenta, vaginal fluids and faeces in women and their babies (nasopharyngeal/throat swabs, blood, saliva, faeces).
- To assess the effects of interventions to prevent COVID-related complications.
- To determine if the rates of prevalence, clinical manifestations, outcomes and risk factors vary by: a) screening and testing strategy for SARS-CoV-2, b) selection of populations, c) risk status of the included women, timing of exposure (first, second, third trimester, postpartum), World Bank economic region (low, middle and high income) and quality of studies (low, high).

METHODS

Our systematic review protocol is registered in Prospero (CRD42020178076).[15] We will regularly repeat the searches, data extraction and analyses as described in Figure 1.

For peer review only - http://bmjopen.bmj.com/site/about/guidelines.xhtml

Literature search

We will carry out a systematic search on the World Health Organisations (WHO) Database of publications on COVID-19, the EPPI-Centre map of the current evidence on COVID-19, Cochrane databases, China National Knowledge Infrastructure (CNKI), Wanfang and preprint databases (ArXiv, BiorXiv, medRxiv, search.bioPreprint), the reference lists of included studies, relevant systematic reviews and guidelines published by national and international professional societies, COVID-19 research websites,[16,17] and follow blogs dedicated to the identification of primary case reports, case series, observational studies or randomised-controlled trials describing women affected by COVID-19 in pregnancy. We will also link with established groups conducting surveillance and research studies in pregnant women with COVID-19 to access their aggregate study data.[18-20] There will be no language restrictions. The search findings will be exported to Covidence (http://covidence.org/), an online programme which facilitates study selection and screening, recommended by the Cochrane Collaboration.[21] Our search will be updated weekly. We shall review this frequency every two months.

Study selection, quality assessment and data extraction

Study selection will be a two-stage process: titles and/or abstracts of studies will be screened first, followed by evaluation of full texts for eligibility by two independent reviewers. Disagreements will be resolved through discussion or by consulting a third reviewer. We will include all cohort studies (with at least 10 participants) and randomised trials reporting on clinical outcomes relating to SARS-CoV-2 infection involving pregnant and postpartum (which includes post-miscarriage/abortion period) women and their babies. We defined cohort studies as those that included women based on exposure, followed-up over specified

For peer review only - http://bmjopen.bmj.com/site/about/guidelines.xhtml

time and reported outcomes.[22] Case reports and case series will only be included to answer the research questions relating to mother-to-child transmission.

We will look for serological (IgM and IgG) and/or RT-PCR (reverse transcriptase polymerase chain reaction) confirmation of infection in amniotic fluid, placenta, cord, newborn and maternal blood, and newborn and maternal respiratory secretions (at birth, 24 and ≥48 hours after birth) to distinguish between congenital, intrapartum and postpartum transmission. Women with a respiratory sample (naso/oropharyngeal swab or tracheal/bronchoalveolar lavage) positive for SARS-CoV-2 RT-PCR or positive SARS-CoV-2 serological tests will be considered to have confirmed COVID-19. We will also assess risk factors such as mode of delivery, rooming-in and breast feeding on mother-to-child transmission. Women with a clinical diagnosis based on chest computerised tomography (CT) or radiograph or other features will be considered as suspected COVID-19. Table 1 provides the details of risk factors, maternal, and perinatal outcomes that will be evaluated in the review.

For peer review only - http://bmjopen.bmj.com/site/about/guidelines.xhtml
For peer review only

Table 1. Study participants, risk factors and outcomes evaluated in the living systematic review on COVID-19 in pregnant and postpartum women

**Population**	Pregnant/ postpartum/ postabortal women with suspected of confirmed COVID-19 infection
**Risk factors**	**Maternal**<br>Age, ethnicity, pre-existing medical conditions (diabetes, chronic hypertension), gestational diabetes, symptoms, hypertensive disorders in pregnancy (pre-eclampsia, pregnancy induced hypertension), BMI (Body Mass Index) ≥ 30, multiple pregnancy, in vitro fertilisation, parity, gestational age, mode of delivery, pregnancy status (pregnant or delivered), Reproductive tract infections
**Clinical manifestations**	**Symptoms and signs**<br>Cough, fever, breathlessness, sputum, myalgia, fatigue, diarrhoea, headache, sore throat, chest pain, rigor, ageusia, anosmia, nausea or vomiting, Sequential Organ Failure Assessment (SOFA), Quick SOFA (qSOFA), asymptomatic<br>**Laboratory**<br>White cell count, lymphocyte count, haemoglobin, anaemia, platelet count, albumin, ALT, AST, C-reactive protein, creatinine, lactate dehydrogenase, creatinine kinase, high-sensitivity cardiac troponin, prothrombin time, D-dimer, serum, ferritin, interleukin-6, procalcitonin<br>**Radiological**<br>Consolidation, ground-glass opacity, bilateral pulmonary infiltration, unilateral pulmonary infiltration, abnormal chest X-Ray, abnormal chest CT
**Outcomes**	**Maternal COVID-related outcomes**<br>Mortality: all-cause mortality, COVID-specific mortality<br>Clinical Respiratory Syndrome: pneumonia, respiratory failure, ARDS (Acute Respiratory Distress Syndrome), severe pneumonia; invasive ventilation, non-invasive ventilation, oxygenation; long-term respiratory outcomes<br>Time from illness onset to outcome (death, recovery)<br>Hospitalisation: admission to ICU (Intensive Care Unit), admission to hospital, ICU length of stay<br>Organ Failure: sepsis, septic shock, cardiac failure, coagulopathy, acute cardiac injury, acute kidney injury, acute hepatic failure, cytokine storm syndrome (haemophagocytic lymphohistiocytosis), hypoproteinaemia, acidosis, central nervous system manifestations, secondary infection, duration of viral shedding<br>Delirium, acute neuropsychiatric emergency, agitation, anxiety, depression, psychosis<br>**Pregnancy-related outcomes**<br>preterm delivery (<37w, spontaneous preterm delivery, induced preterm birth), preterm delivery, preterm-premature rupture of membranes, prelabour rupture of membranes at (or near) term, miscarriage (spontaneous), induced abortion, mode of delivery, induction of labour, chorioamnionitis, wound infection, pregnancy-induced hypertension, gestational diabetes, antepartum haemorrhage, postpartum haemorrhage<br>**Offspring outcomes**<br>Stillbirth, neonatal death (early, late), foetal distress, foetal growth restriction post infection, Apgar score at 1’, 5’; cord blood pH, gestational age at delivery, birthweight, LGA (large-for-gestational age), SGA (small-for-gestational age), congenital malformation, HIE (hypoxic ischaemic encephalopathy), neonatal seizures, neonatal infection (other than COVID), neonatal sepsis, neonatal asphyxia, DIC (Disseminated Intravascular Coagulation), NEC (Necrotising Enterocolitis), RDS (Respiratory Distress Syndrome), admission to the neonatal unit, length of stay in neonatal unit<br>**Mother-to-child transmission outcomes**<br>Evidence of virus in amniotic fluid, cord blood, placenta, placental membranes, vaginal fluid, breast milk, neonatal throat swabs, maternal and neonatal faeces and saliva samples; IgM antibodies in cord blood, neonatal blood<br>Duration of viral shedding after COVID-19 symptoms onset and after clinical resolution of signs/symptoms in mother and in newborn

For peer review only - http://bmjopen.bmj.com/site/about/guidelines.xhtml

We will assess the risk of bias for included cohort studies using the Newcastle Ottawa Scale for comparative cohorts,[23] the Cochrane Risk of Bias 2 (RoB 2) tool for randomised trials,[24] QUADAS-2 for diagnostic accuracy studies,[25] and the tool outlined by Hoy et al. for prevalence studies.[26] A pre-piloted form will be used for data extraction of the included studies. Two reviewers will independently extract data and disagreements will be resolved by consensus or by consulting a third reviewer. We will assess for duplication of the data by comparing characteristics of the mother or baby and the settings of the studies. If required, we will contact the authors of primary studies for clarification about duplicate data. Where possible, we have planned a semi-automated process to identify duplicates and facilitate rapid update of search and data extraction.

Analysis

We will undertake narrative syntheses and perform aggregate meta-analyses when there are at least two studies with minimal clinical heterogeneity. Dichotomous outcomes will be summarised as proportions, odds ratios (OR) and continuous outcomes as standardised mean differences. We will use random effects model for the analysis when the number of studies permits to estimate between-study variance. To summarise proportions, we will use Freeman-Tukey transformation to stabilize variances while dealing with studies with zero events. We will provide 95% confidence intervals (CI) and predictive intervals (PI) to report on the precision of estimates and to aid the interpretation of heterogeneity. Heterogeneity will be reported as I^2 and tau-squared statistics.

Pre-planned subgroup analysis will be by a) suspected/probable or confirmed COVID-19, b) diagnosis in pregnancy or postnatal period, c) trimester of diagnosis (first, second or third), d) country income-level (high or low- and middle-income country), e) screening strategies

For peer review only - http://bmjopen.bmj.com/site/about/guidelines.xhtml

(universal, symptom-based or risk-based testing) and f) maternal risk status (low or high). We will undertake additional sensitivity analysis to explore the effects of different populations (unselected, selected) and by excluding women with suspected COVID-19, studies with overlapping samples and studies at high risk of bias. We will use trial sequential analysis to control for type I and II errors while accounting for updating estimations of between-study heterogeneity. A priori assumptions on statistical power, minimal clinically relevant effect and heterogeneity between trials will be used to define maximum sample size to detect such an effect. Boundaries of statistical significance according to sample size will be defined and used to determine statistical significance for each systematic review update result.

We have established a pool of peer reviewers to rapidly assess the findings. The initial pre-peer reviewed findings will be published in a dedicated website, followed by the full findings when peer review is complete. We will simultaneously submit our work for publication in scientific journals with clear reference to the version of the living systematic review provided in that submission. Where journals allow, we will update our findings in the journals at set intervals required by the journal.

Patient and public involvement

Katie's Team Patients and Public Involvement group- a dedicated women's research and health advisory group, were involved in the design of the protocol, and will contribute to the interpretation and dissemination of the result.

DISCUSSION

Our living systematic review will address key research questions relevant to SARS-CoV-2 infection in pregnancy and postpartum period. Our review is based on a prospective protocol

For peer review only

For peer review only - http://bmjopen.bmj.com/site/about/guidelines.xhtml

with plans to continuously update all review processes from search to publication at specific time points. The findings of the review will directly inform living guidelines and policies as new evidence emerge.

Our detailed literature search is a major strength of the review. In addition to the WHO COVID-19 database, our links with Cochrane Fertility and Gynaecology and EPPI Centre groups, and additional searches for studies mapped by dedicated websites as well as blogs and social media networks means that the chance of missing relevant studies are small. Through our networks with key collaborators in the WHO MNCAH (Maternal, neonatal, child and adolescent health) COVID-19 research group,[27] and relevant working groups, we will be able to access and include unpublished data. Our collaborative links with researchers in China provides access to Chinese language databases, so that these studies are not missed.

We have developed the protocol to ensure that all stages of the review are robust by adhering to recommended methods for conduct and reporting of systematic reviews.[28] By restricting case reports and series to the research question on mother-to-child transmission alone, we will not only ensure that relevant cases are not missed, but also avoid biased estimates for other research questions on risk factors and prevalence. Determination of mother-to-child SARS-CoV-2 transmission is particularly challenging since there is no consensus on what constitutes intrauterine, intrapartum, and postpartum transmission (including breast-milk versus horizontal transmission).[6] To maximise data collection addressing this question, we will include case reports and case series for this part of the review. We will collect data on all types of samples to enable evaluation of varying definitions of the evidence required for confirmation of mother-to-child SARS-CoV-2 transmission and timing.

For peer review only - http://bmjopen.bmj.com/site/about/guidelines.xhtml

Our work is subject to some limitations. Our protocol has been developed based on our current knowledge of the COVID-19. As the pandemic unfolds, emerging new evidence may require changes in the protocol. The systematic review will be influenced by the characteristics of the individual studies, which may comprise heterogeneous populations, definitions of COVID-19, sampling frames, test strategies for diagnosis, definitions and reporting of outcomes. We have addressed these challenges by clearly defining the inclusion criteria, and by exploring heterogeneity through sensitivity and subgroup analyses. The findings will be reported as 95% CI to communicate the uncertainty around the pooled estimates and prediction intervals (PI) to anticipate the variability in new study estimates. Given the urgency of the situation in the pandemic era, many studies are published as preprints, often followed by a full publication at a later date. The living systematic review needs to be responsive to any changes in data between the preprint to full publication stage. Furthermore, there is a risk of duplicate data, as individual studies may report the findings of the same mother-baby data that have been published elsewhere. We plan to exclude studies with suspected duplicate data, undertake sensitivity analyses where required, and contact the authors of the primary studies for clarification. This review may be subject to publication bias, since studies with perceived positive results may be published faster than those with perceived negative results. We will continuously review registries of randomised controlled trials to detect trials that should have reported results but that have not done so and will contact corresponding authors to obtain results.

Many of the automated tools for living systematic review are mainly developed for reviews on randomised trials (RCT), and not for observational studies.[29] Unlike traditional systematic reviews, living systematic reviews require substantial investment in time and human resources, and resources to regularly update the findings. Sustained funding is required to

For peer review only - http://bmjopen.bmj.com/site/about/guidelines.xhtml

maintain the same level of output over a longer period of time. This is particularly challenging, as the publication rate of studies on SARS-Cov-2 infection in pregnancy and postpartum is likely to increase exponentially. To ensure that all relevant studies are identified in a timely manner, in addition to traditional searches, we will use our collaborations with other global efforts to map studies on COVID-19 and pregnancy and postpartum, and automated alerts to identify new evidence when it gets published. Given the wide scope of this review, numerous reviewers will be involved, requiring clear operating procedures and pathways in place for workflow, training, monitoring and quality assessment. The necessity to swiftly publish the collated evidence need to be balanced against publishing the findings after peer review. To be able to sustain the level of reviewer turn-around every two weeks will be a challenge.

To-date, apart from Cochrane, very few journals provide specific guidelines for publication of living systematic reviews and its subsequent update.[30] Various models of publication have been considered.[31] The first model is similar to the Cochrane reviews, where with each new update (usually done in yearly intervals), there is a new publication with a new version and DOI in PubMed with linked updates between versions. This model can also take into account any changes in authors between the versions.[32] However, in the current situation of rapidly evolving evidence, this is likely to result in numerous publications, even if reported on a monthly basis. In the second model, the introduction and methods of the main manuscript does not change, with only changes in the results section, which is written in such a way that most of the information is provided in Tables and Figures that are revised along with the abstract. The discussion section of the newer version can incorporate a paragraph on the implications of new findings. The manuscript will be less resource intensive to prepare in the second model, but the original version should have been written in a generic manner to

For peer review only

accommodate new information emerging in subsequent versions. In another model, the findings of subsequent updated analyses appear as new appendices, with no changes in the original abstract or manuscript. While this model requires less efforts from authors, editors and peer reviewers, it can mistakenly provide inaccurate old evidence if readers only access the abstract. Furthermore, this model makes it difficult to add or remove authors according to the changes in their contribution to subsequent versions.

In the midst of the current pandemic, much still remains unknown about how SARS-CoV-2 infection affects pregnant and postpartum women and their babies compared to reproductive aged non-pregnant women. It is essential that clinicians' decisions to manage pregnant and postpartum women and their babies with COVID-19 are guided by the evidence. Our living systematic review is well suited to rapidly provide updated findings for translation into clinical practice. The flexibility of the living systematic review needs to be matched by a willingness of journal editors and guideline makers to provide a framework that allows rapid dissemination of the new findings.

For peer review only

Ethics and dissemination

No ethical concerns. The findings will be disseminated through a designated website, publications, presentations in webinars and social media.

Data statement

Data sharing not applicable as no datasets generated and/or analysed for this study

Author contributions

All authors contributed to the development of the protocol and writing of the manuscript.

MY, LD, TK, SC - wrote the first draft of the manuscript and are involved in study selection and data extraction, and have approved the final version of the manuscript.

For peer review only - http://bmjopen.bmj.com/site/about/guidelines.xhtml

JA, ES - designed the study, and are involved in data extraction and analysis. They reviewed the manuscript and have approved the final version of the manuscript.

DC, SL - reviewed the manuscript and are involved in quality assessment and data extraction. They have approved the final version of the manuscript.

XQ, MY, MvW, EK, EvL, LM, HK, AK, ST, JT, VB, NB, ED, CRK, AT, OO - reviewed the manuscript, contributing critical changes, and they have approved the final version of the manuscript.

ACL, AD, DZ, RB - reviewed the manuscript and are involved in study selection, and they have approved the final version of the manuscript.

JZ - reviewed the manuscript and will conduct the statistical analysis. He approved the final version of the manuscript.

MB - designed the study, and reviewed the manuscript, and has approved the final version of the manuscript.

ST - conceived and designed the study, and reviewed the manuscript and approved the final version of the manuscript. ST is the guarantor.

Funding statement

The work is partially funded by the World Health Organization. The authors alone are responsible for the views expressed in this manuscript and they do not necessarily represent the decisions, policy or views of the funding bodies. The views of the funding bodies have not influenced the content of this manuscript.

Competing interests

None

References

1. WHO announces COVID-19 outbreak a pandemic: World Health Organisation [Internet]; 2020. https://www.euro.who.int/en/health-topics/health-emergencies/coronavirus-covid-19/news/news/2020/3/who-announces-covid-19-outbreak-a-pandemic (accessed Jun 11 2020).
2. COVID-19 Dashboard: John Hopkins University [Internet]. https://coronavirus.jhu.edu/map.html (accessed Apr 21 2020).
3. Rajgor DD, Lee MH, Archuleta S, et al. The many estimates of the COVID-19 case fatality rate. *Lancet Infect Dis* 2020 doi: 10.1016/S1473-3099(20)30244-9
4. Mor G, Cardenas I. The immune system in pregnancy: a unique complexity. *Am J Reprod Immunol* 2010;63(6):425-33. doi: 10.1111/j.1600-0897.2010.00836.x
5. Schwartz DA, Graham AL. Potential Maternal and Infant Outcomes from (Wuhan) Coronavirus 2019-nCoV Infecting Pregnant Women: Lessons from SARS, MERS, and Other Human Coronavirus Infections. *Viruses* 2020;12(2) doi: 10.3390/v12020194

For peer review only - http://bmjopen.bmj.com/site/about/guidelines.xhtml

6. Blumberg DA, Underwood MA, Hedriana HL, et al. Vertical Transmission of SARS-CoV-2: What is the Optimal Definition? *Am J Perinatol* 2020 doi: 10.1055/s-0040-1712457
7. Covid-19 and pregnancy. *BMJ* 2020;369:m1672. doi: 10.1136/bmj.m1672
8. Kmietowicz Z. Covid-19: Half of pregnant women in UK hospitals are from ethnic minorities. *BMJ* 2020;369:m2266. doi: 10.1136/bmj.m2266
9. Millett GA, Jones AT, Benkeser D, et al. Assessing Differential Impacts of COVID-19 on Black Communities. *Ann Epidemiol* 2020 doi: 10.1016/j.annepidem.2020.05.003
10. Knight M, Bunch K, Vousden N, et al. Characteristics and outcomes of pregnant women admitted to hospital with confirmed SARS-CoV-2 infection in UK: national population based cohort study. *BMJ* 2020;369:m2107. doi: 10.1136/bmj.m2107
11. Haghani M, Bliemer MCJ, Goerlandt F, et al. The scientific literature on Coronaviruses, COVID-19 and its associated safety-related research dimensions: A scientometric analysis and scoping review. *Saf Sci* 2020:104806. doi: 10.1016/j.ssci.2020.104806
12. Lee AY, Lin MW. Rapid publishing in the era of coronavirus disease 2019 (COVID-19). *Med J Aust* 2020 doi: 10.5694/mja2.50617
13. Knottnerus JA, Tugwell P. Methodological challenges in studying the COVID-19 pandemic crisis. *J Clin Epidemiol* 2020;121:A5-A7. doi: 10.1016/j.jclinepi.2020.04.001
14. Shokraneh F, Adams CE. Study-based registers of randomized controlled trials: Starting a systematic review with data extraction or meta-analysis. *Bioimpacts* 2017;7(4):209-17. doi: 10.15171/bi.2017.25
15. Allotey J, Bonet M, Zamora J, et al. COVID-19 in pregnant women: a Living Systematic Review on prevalence, presentation, prognosis and treatment. PROSPERO 2020 CRD42020178076. *Prospero [Internet]* 2020
16. COVID-19, Maternal and Child Health, Nutrition: John Hopkins Center for Humanitarian health [Internet]. http://hopkinshumanitarianhealth.org/empower/advocacy/covid-19/covid-19-children-and-nutrition/ (accessed Jun 9 2020).
17. COVID-19 Research Community: ResearchGate [Internet]. https://www.researchgate.net/community/COVID-19 (accessed Jun 9 2020).
18. COVID-19: a living systematic map of the evidence: Eppi-centre [Internet]. http://eppi.ioe.ac.uk/cms/Projects/DepartmentofHealthandSocialCare/Publishedreviews/COVID-19Livingsystematicmapoftheevidence/tabid/3765/Default.aspx (accessed Jun 11 2020).
19. NIPH systematic and living map on COVID-19 evidnce: Norwegian Institute of Public Health [Internet]. https://www.nornesk.no/forskningskart/NIPH_mainMap.html (accessed Jun 11 2020).
20. Advanced Search: Cochrane Library. https://www.cochranelibrary.com/advanced-search (accessed Jun 11 2020).
21. Covidence: Cochrane. https://community.cochrane.org/help/tools-and-software/covidence (accessed Jun 14 2020).
22. Dekkers OM, Egger M, Altman DG, et al. Distinguishing case series from cohort studies. *Ann Intern Med* 2012;156(1 Pt 1):37-40. doi: 10.7326/0003-4819-156-1-201201030-00006
23. Wells G, Shea B, O'Connell D, et al. The Newcastle-Ottowa Scale (NOS) for assessing the quality of nonrandomised studies in meta-analysis: The Ottowa Hospital Research Institute; 2013. http://www.ohri.ca/programs/clinical_epidemiology/oxford.asp (accessed Jun 11 2020).
24. Sterne JAC, Savovic J, Page MJ, et al. RoB 2: a revised tool for assessing risk of bias in randomised trials. *BMJ* 2019;366:l4898. doi: 10.1136/bmj.l4898

For peer review only

25. Whiting PF, Rutjes AW, Westwood ME, et al. QUADAS-2: a revised tool for the quality assessment of diagnostic accuracy studies. *Ann Intern Med* 2011;155(8):529-36. doi: 10.7326/0003-4819-155-8-201110180-00009
26. Hoy D, Brooks P, Woolf A, et al. Assessing risk of bias in prevalence studies: modification of an existing tool and evidence of interrater agreement. *J Clin Epidemiol* 2012;65(9):934-9. doi: 10.1016/j.jclinepi.2011.11.014
27. Maternal, newborn, child and adolescent health (MNCAH) consolidated data portal: World Health Organisation [Internet]. https://www.who.int/maternal_child_adolescent/epidemiology/mncah-data-platform/en/ (accessed Jun 11 2020).
28. Moher D, Liberati A, Tetzlaff J, et al. Preferred reporting items for systematic reviews and meta-analyses: the PRISMA statement. *BMJ* 2009;339:b2535. doi: 10.1136/bmj.b2535
29. Khamis AM, Kahale LA, Pardo-Hernandez H, et al. Methods of conduct and reporting of living systematic reviews: a protocol for a living methodological survey. *F1000Res* 2019;8:221. doi: 10.12688/f1000research.18005.2
30. Brooker J, Synnot A, Mcdonald S, et al. Guidance for the production and publication of Cochrane living systematic reviews: Cochrane Reviews in living mode. 2019. https://community.cochrane.org/sites/default/files/uploads/inline-files/Transform/201912_LSR_Revised_Guidance.pdf.
31. Elliott JH, Synnot A, Turner T, et al. Living systematic review: 1. Introduction-the why, what, when, and how. *J Clin Epidemiol* 2017;91:23-30. doi: 10.1016/j.jclinepi.2017.08.010
32. Cumptsom M, Chandler J. Updating a review. In: Higgins JPT TJ, Chandler J, Cumpston M, Li T, Page MJ, Welch VA, ed. Cochrane Handbook for Systematic Reviews of Interventions version 60 (updated August 2019): Cochrane, 2019.

Figure Legend

Figure 1: Steps in the living systematic review (LSR) on COVID-19 in pregnant and postpartum women

For peer review only

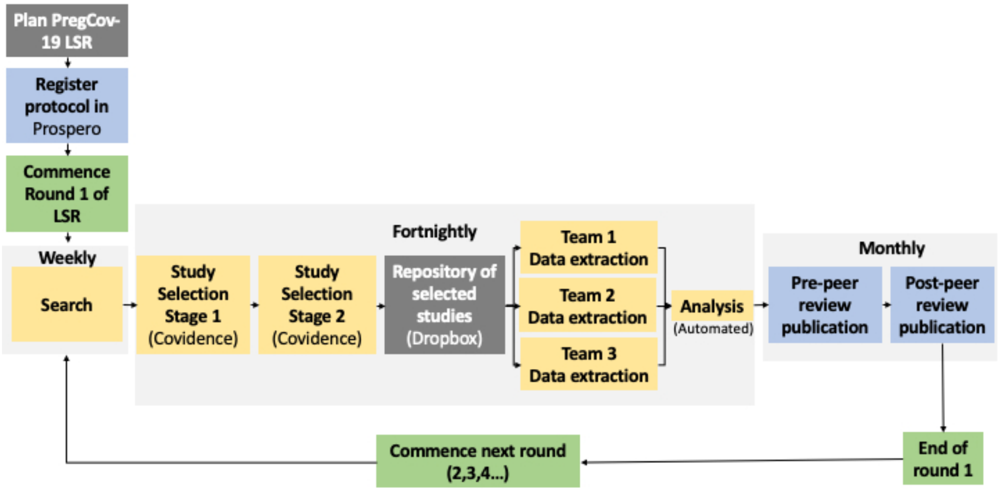

Fig 1. Steps in the living systematic review (LSR) on COVID-19 in pregnant and postpartum women

338x190mm (54 x 54 DPI)

PRISMA-P (Preferred Reporting Items for Systematic review and Meta-Analysis Protocols) 2015 checklist addressed in the living systematic review protocol on COVID-19 in pregnant and recently pregnant women

Section and topic	Item No	Checklist item	Page No.
**ADMINISTRATIVE INFORMATION**			
Title:			
Identification	1a	Identify the report as a protocol of a systematic review	1
Update	1b	If the protocol is for an update of a previous systematic review, identify as such	Not applicable
Registration	2	If registered, provide the name of the registry (such as PROSPERO) and registration number	4
Authors:			
Contact	3a	Provide name, institutional affiliation, e-mail address of all protocol authors; provide physical mailing address of corresponding author	1-2
Contributions	3b	Describe contributions of protocol authors and identify the guarantor of the review	16
Amendments	4	If the protocol represents an amendment of a previously completed or published protocol, identify as such and list changes; otherwise, state plan for documenting important protocol amendments	Not applicable
Support:			
Sources	5a	Indicate sources of financial or other support for the review	16
Sponsor	5b	Provide name for the review funder and/or sponsor	16
Role of sponsor or funder	5c	Describe roles of funder(s), sponsor(s), and/or institution(s), if any, in developing the protocol	16
**INTRODUCTION**			
Rationale	6	Describe the rationale for the review in the context of what is already known	6-7
Objectives	7	Provide an explicit statement of the question(s) the review will address with reference to participants, interventions, comparators, and outcomes (PICO)	7-8
**METHODS**			
Eligibility criteria	8	Specify the study characteristics (such as PICO, study design, setting, time frame) and report characteristics (such as years considered, language, publication status) to be used as criteria for eligibility for the review	9

For peer review only

Information sources	9	Describe all intended information sources (such as electronic databases, contact with study authors, trial registers or other grey literature sources) with planned dates of coverage	9
Search strategy	10	Present draft of search strategy to be used for at least one electronic database, including planned limits, such that it could be repeated	9
Study records:			
Data management	11a	Describe the mechanism(s) that will be used to manage records and data throughout the review	9
Selection process	11b	State the process that will be used for selecting studies (such as two independent reviewers) through each phase of the review (that is, screening, eligibility and inclusion in meta-analysis)	9
Data collection process	11c	Describe planned method of extracting data from reports (such as piloting forms, done independently, in duplicate), any processes for obtaining and confirming data from investigators	9
Data items	12	List and define all variables for which data will be sought (such as PICO items, funding sources), any pre-planned data assumptions and simplifications	10
Outcomes and prioritization	13	List and define all outcomes for which data will be sought, including prioritization of main and additional outcomes, with rationale	10
Risk of bias in individual studies	14	Describe anticipated methods for assessing risk of bias of individual studies, including whether this will be done at the outcome or study level, or both; state how this information will be used in data synthesis	10
Data synthesis	15a	Describe criteria under which study data will be quantitatively synthesised	11
	15b	If data are appropriate for quantitative synthesis, describe planned summary measures, methods of handling data and methods of combining data from studies, including any planned exploration of consistency (such as I^2, Kendall's τ)	11
	15c	Describe any proposed additional analyses (such as sensitivity or subgroup analyses, meta-regression)	11
	15d	If quantitative synthesis is not appropriate, describe the type of summary planned	11
Meta-bias(es)	16	Specify any planned assessment of meta-bias(es) (such as publication bias across studies, selective reporting within studies)	11
Confidence in cumulative evidence	17	Describe how the strength of the body of evidence will be assessed (such as GRADE)	11

For peer review only

BMJ Open

Clinical manifestations, prevalence, risk factors, outcomes, transmission, diagnosis and treatment of coronavirus disease (COVID-19) in pregnancy and postpartum: A living systematic review protocol

Journal:	*BMJ Open*
Manuscript ID	bmjopen-2020-041868.R1
Article Type:	Protocol
Date Submitted by the Author:	03-Sep-2020

Complete List of Authors:	Yap, Magnus; University of Birmingham College of Medical and Dental Sciences<br>Debenham, Luke; University of Birmingham College of Medical and Dental Sciences<br>Kew, Tania; University of Birmingham College of Medical and Dental Sciences<br>Chatterjee, Shaunak; University of Birmingham College of Medical and Dental Sciences<br>Allotey, John; University of Birmingham Institute of Applied Health Research; University of Birmingham, WHO Collaborating Centre for Global Women's Health, Institute of Metabolism and Systems Research<br>Stallings, Elena; IRYCIS, Clinical Biostatistics Unit; CIBERESP<br>Coomar, Dyuti; University of Birmingham Institute of Applied Health Research<br>Lee, Siang; University of Birmingham Institute of Applied Health Research<br>Qiu, xiu; Guangzhou Women and Children's Medical Center, Division of Birth Cohort Study; Guangzhou Women and Children's Medical Center, Department of Obstetrics and Gynecology<br>Yuan, Mingyang; Guangzhou Women and Children's Medical Center, Division of Birth Cohort Study<br>Clavé Llavall, Anna; University of Birmingham College of Medical and Dental Sciences<br>Dixit, Anushka; University of Birmingham College of Medical and Dental Sciences<br>Zhou, Dengyi; University of Birmingham College of Medical and Dental Sciences<br>Balaji, Rishab; University of Birmingham College of Medical and Dental Sciences<br>van Wely, Madelon; Amsterdam University Medical Centres, Netherlands Satellite of the Cochrane Gynaecology and Fertility Group<br>Kostova, Elena; Amsterdam University Medical Centres, Netherlands Satellite of the Cochrane Gynaecology and Fertility Group<br>van Leeuwen, Elisabeth; Amsterdam University Medical Centres, Department of Obstetrics and Gynaecology<br>Mofenson, Lynne; Elizabeth Glaser Pediatric AIDS Foundation,<br>Kunst, Heinke; Queen Mary University of London, Department of Respiratory Medicine

	Khalil, Asma; St George's University of London<br>Tiberi, Simon; Barts Health NHS Trust, Blizard Institute, Queen Mary University of London, Department of Infection; Barts Health NHS Trust, Department of Infectious Diseases<br>Thomas, James; University College London Social Science Research Unit<br>Brizuela, Vanessa; WHO, Department of Reproductive Health and Research<br>Broutet, Nathalie; WHO, Department of Reproductive Health and Research<br>Kara, Edna; WHO, Department of Sexual and Reproductive Health and Research<br>Kim, Caron; WHO, Department of Sexual and Reproductive Health and Research<br>Thorson, Anna; WHO, Department of Sexual and Reproductive Health and Research<br>Rayco-Solon, Pura; WHO, The Department of Maternal, Newborn, Child and Adolescent Health<br>Pardo-Hernandez, Hector; WHO, Cross-cutting Global Initiatives/Nutrition and Food Safety department<br>Oladapo, Olufemi ; WHO, Department of Sexual and Reproductive Health and Research<br>Zamora , Javier ; IRYCIS, Clinical Biostatistics Unit; CIBERESP<br>Bonet, Mercedes; WHO, Department of Sexual and Reproductive Health and Research<br>Thangaratinam, Shakila; Birmingham Women's and Children's Hospitals NHS Foundation Trust; University of Birmingham, WHO Collaborating Centre for Global Women's Health, Institute of Metabolism and Systems Research
**Primary Subject Heading**:	Obstetrics and gynaecology
Secondary Subject Heading:	Infectious diseases, Research methods, Epidemiology
Keywords:	Maternal medicine < OBSTETRICS, VIROLOGY, NEONATOLOGY, Protocols & guidelines < HEALTH SERVICES ADMINISTRATION & MANAGEMENT, PERINATOLOGY

SCHOLARONE™
Manuscripts

I, the Submitting Author has the right to grant and does grant on behalf of all authors of the Work (as defined in the below author licence), an exclusive licence and/or a non-exclusive licence for contributions from authors who are: i) UK Crown employees; ii) where BMJ has agreed a CC-BY licence shall apply, and/or iii) in accordance with the terms applicable for US Federal Government officers or employees acting as part of their official duties; on a worldwide, perpetual, irrevocable, royalty-free basis to BMJ Publishing Group Ltd ("BMJ") its licensees and where the relevant Journal is co-owned by BMJ to the co-owners of the Journal, to publish the Work in this journal and any other BMJ products and to exploit all rights, as set out in our licence.

The Submitting Author accepts and understands that any supply made under these terms is made by BMJ to the Submitting Author unless you are acting as an employee on behalf of your employer or a postgraduate student of an affiliated institution which is paying any applicable article publishing charge ("APC") for Open Access articles. Where the Submitting Author wishes to make the Work available on an Open Access basis (and intends to pay the relevant APC), the terms of reuse of such Open Access shall be governed by a Creative Commons licence – details of these licences and which Creative Commons licence will apply to this Work are set out in our licence referred to above.

Other than as permitted in any relevant BMJ Author's Self Archiving Policies, I confirm this Work has not been accepted for publication elsewhere, is not being considered for publication elsewhere and does not duplicate material already published. I confirm all authors consent to publication of this Work and authorise the granting of this licence.

For peer review only

Clinical manifestations, prevalence, risk factors, outcomes, transmission, diagnosis and treatment of coronavirus disease (COVID-19) in pregnancy and postpartum: A living systematic review protocol

Magnus Yap*,[1] Luke Debenham*,[1] Tania Kew*,[1] Shaunak Chatterjee*,[1] John Allotey,[2,3] Elena Stallings,[4,5] Dyuti Coomar,[2] Siang Ing Lee,[2] Xiu Qiu,[6,7,8] Mingyang Yuan,[2,6], Anna Clavé Llavall,[1] Anushka Dixit,[1] Dengyi Zhou,[1] Rishab Balaji,[1] Madelon van Wely,[9] Elena Kostova,[9] Elisabeth van Leeuwen,[10] Lynne Mofenson,[11] Heinke Kunst,[12] Asma Khalil,[13] Simon Tiberi,[12,14] James Thomas,[15] Vanessa Brizuela,[16] Nathalie Broutet,[16] Edna Kara,[16] Caron Rahn Kim,[16] Anna Thorson,[16] Pura Rayco-Solon,[16] Hector Pardo-Hernandez,[16] Olufemi Taiwo Oladapo,[16] Javier Zamora,[4,5,17] Mercedes Bonet,[16] Shakila Thangaratinam[3,18] for PregCOV-19 Consortium

[1]Birmingham Medical School, University of Birmingham, Birmingham, United Kingdom
[2]Institute of Applied Health Research, University of Birmingham, Birmingham, United Kingdom
[3]WHO Collaborating Centre for Global Women's Health, Institute of Metabolism and Systems Research, University of Birmingham, Birmingham, United Kingdom
[4]Clinical Biostatistics Unit, Hospital Universitario Ramón y Cajal (IRYCIS), Madrid, Spain
[5]CIBER Epidemiology and Public Health (CIBERESP), Madrid, Spain
[6]Division of Birth Cohort Study, Guangzhou Women and Children's Medical Center, Guangzhou Medical University, Guangzhou, China
[7]Department of Woman and Child Health Care, Guangzhou Women and Children's Medical Center, Guangzhou Medical University, Guangzhou, China
[8]Department of Obstetrics and Gynecology, Guangzhou Women and Children's Medical Center, Guangzhou Medical University, Guangzhou, China
[9]Netherlands Satellite of the Cochrane Gynaecology and Fertility Group, Amsterdam University Medical Center, Amsterdam, The Netherlands
[10]Department of Obstetrics and Gynaecology, Amsterdam University Medical Center, Amsterdam, The Netherlands
[11]Elizabeth Glaser Pediatric AIDS Foundation, Washington DC, United States
[12]Blizard Institute, Queen Mary University of London, London, United Kingdom
[13]St George's University London, London, United Kingdom
[14]Department of Infectious Diseases, Barts Health NHS Trust, London, United Kingdom
[15]UCL Institute of Education, University College London, London, United Kingdom
[16]UNDP/UNFPA/UNICEF/WHO/World Bank Special Programme of Research, Development and Research Training in Human Reproduction (HRP), Department of Sexual and Reproductive Health and Research, World Health Organization, Avenue Appia 20, 1211, Geneva, Switzerland
[17]Women's Health Research Unit, Queen Mary University of London, London, United Kingdom
[18]Birmingham Women's and Children's NHS Foundation Trust, Birmingham, United Kingdom

*Joint first authors

For peer review only

Corresponding author: John Allotey, Institute of Applied Health Research, University of Birmingham, College of Medical and Dental Sciences, Birmingham, United Kingdom.
Email: j.allotey.1@bham.ac.uk

Word count: 2783

Keywords: COVID-19, pregnancy, postpartum, neonate, living systematic review, meta-analysis, prevalence, risk factors, outcomes, vertical transmission, diagnosis, diagnostic tests, treatment

For peer review only

Abstract

Introduction

Rapid, robust and continually updated evidence synthesis is required to inform management of COVID-19 (coronavirus disease) in pregnant and postpartum women, and keep pace with the emerging evidence during the pandemic.

Methods and analysis

We plan to undertake a living systematic review to assess the prevalence, clinical manifestations, risk factors, rates of maternal and perinatal complications, potential for mother-to-child transmission, accuracy of diagnostic tests, and effectiveness of treatment for COVID-19 in pregnant and postpartum women (including after miscarriage or abortion). We will search Medline, Embase, World Health Organization COVID-19 database, preprint servers, the China National Knowledge Infrastructure system and Wanfang databases from 1 December 2019. We will supplement our search with studies mapped by Cochrane Fertility and Gynaecology group, EPPI-Centre, COVID-19 study repositories, reference lists, and social media blogs. The search will be updated every week and not be restricted by language.

We will include observational cohort (≥10 participants) and randomised studies reporting on prevalence of COVID-19 in pregnant and postpartum women, and the rates of clinical manifestations and outcomes, and risk factors in pregnant and postpartum women alone or in comparison with non-pregnant women with COVID-19 or pregnant women without COVID-19, and studies on tests and treatments for COVID-19. We will additionally include case reports and series with evidence on mother-to-child transmission of SARS-CoV-2 in utero, intrapartum or postpartum.

For peer review only - http://bmjopen.bmj.com/site/about/guidelines.xhtml
For peer review only

We will appraise the quality of the included studies using appropriate tools to assess the risk of bias. At least two independent reviewers will undertake study selection, quality assessment and data extraction every two weeks. We will synthesise the findings using quantitative random effects meta-analysis and report odds ratios (OR) or proportions with 95% confidence intervals and prediction intervals. Case reports and series will be reported as qualitative narrative synthesis. Heterogeneity will be reported as I^2 and tau-squared statistics.

Word count – 300

Ethics and dissemination: Ethical approval is not required as this is a synthesis of primary data. Regular updates of the results will be published on a dedicated website (https://www.birmingham.ac.uk/research/who-collaborating-centre/pregcov/index.aspx) and disseminated through publications, social media and webinars.

Protocol registration number on PROSPERO: CRD42020178076

Summary - 'Strengths and limitations of this study'

- Our living systematic review will be underpinned by a comprehensive literature search, study quality assessment, and appropriate planned meta-analysis to efficiently collate the overall findings on COVID-19 in pregnant and postpartum women.
- We will continuously update our search, study selection, data extraction, analysis and reporting of the findings at pre-specified time intervals.
- Rapid publication of new studies with new outcomes of interest, screening and testing strategies and reporting of novel treatments may require changes in the review

For peer review only

protocol, specifically regarding data extraction and search strategies for future updates.

- This review may be subject to publication bias, since studies with perceived positive results may be published faster than those with perceived negative results thus we will continuously review registries of randomised controlled trials to detect trials that should have reported results but that have not done so and will contact corresponding authors to obtain results.
- The dynamic nature of the living systematic review requires dedicated team of committed researchers and resources, efficient peer review and support of the journal editors, to ensure that findings are rapidly published in the public domain with provision for continuous updates to inform living guidelines and policies.

For peer review only

For peer review only - http://bmjopen.bmj.com/site/about/guidelines.xhtml

INTRODUCTION

Coronavirus disease (COVID-19) caused by the severe acute respiratory syndrome coronavirus 2 (SARS-CoV-2) was declared a pandemic by the World Health Organization (WHO) on May 11, 2020.[1] In the first three months of the pandemic alone, over 4.5 million individuals have been affected - the infection continues to spread rapidly.[2] Case fatality rates range from 0.4-3.6% depending on the country and detection method.[3,4] In previous serious coronavirus outbreaks caused by SARS and MERS (Middle East Respiratory Syndrome), the rates of intensive care unit admission and mortality were significantly higher in infected pregnant than non-pregnant women, and adverse pregnancy outcomes were common.[5] There are concerns about the potential effects of SARS-CoV-2 infection on mothers and babies, including the risks of transmission to the foetus and neonate.[6] Some countries such as the UK have classed pregnant women as a vulnerable group requiring shielding during the pandemic.[7] Black and ethnic minority individuals are considered to be more likely to be infected and have severe COVID-19 disease than Caucasian individuals.[8-10]

There has been a rapid increase in the numbers of published studies and reports on the prevalence of SARS-CoV-2 infection in pregnancy, risk factors, mother-to-child transmission, effects on pregnant and recently pregnant women, including those who have delivered or had a recent abortion, and their babies.[11] Traditional systematic reviews are not able to keep pace with the rapid pace of publications, and quickly become outdated.[12] Furthermore, numerous systematic reviews, addressing similar questions and including identical numbers of studies that differ very slightly from each other, make it challenging for guideline makers to identify the up to-date evidence.[13,14] Many of these reviews do not follow reporting guidelines and other general principles of conducting robust systematic

For peer review only - http://bmjopen.bmj.com/site/about/guidelines.xhtml

reviews, often including case series and case control studies in the meta-analysis resulting in biased estimates.

Any recommendation on the care of pregnant women and recently pregnant women with suspected or confirmed COVID-19, and their babies, should be based on robust evidence. Clinicians need a single point of reference that comprehensively provides up-to-date evidence for key questions. In a rapidly changing research and clinical environment, this requires a clear prospective plan to update the available evidence beyond conventional systematic reviews and meta-analyses. We propose to undertake a living systematic review to address the key research questions on SARS-CoV-2 infection in pregnant and postpartum women, including after childbirth and early pregnancy loss.

AIM

Our goal is to provide up to-date evidence on the risks and risk factors for COVID-19 and associated complications in pregnant and postpartum women and their babies through a living systematic review and meta-analysis, and to assess the accuracy of diagnostic tests and effectiveness of treatment in the management of the disease.

OBJECTIVES

In pregnant and postpartum women (including after miscarriage or abortion):

- To determine the prevalence of and risk factors for SARS-CoV-2 infection and severe COVID-19.
- To evaluate the accuracy of tests and prediction models for screening, diagnosis and prediction of COVID-19 and its complications.
- To assess the effects of interventions for prevention of SARS-CoV-2 infection.

For peer review only - http://bmjopen.bmj.com/site/about/guidelines.xhtml

In pregnant and postpartum women (including after miscarriage) with suspected or confirmed COVID-19:

- To study the rates of clinical symptoms and signs, laboratory and radiological manifestations of the disease, and compare against non-pregnant women.
- To assess the rates and risk factors for COVID-related and pregnancy-related maternal and perinatal outcomes and compare against non-pregnant women with COVID-19, and pregnant women without the disease respectively.
- To determine the risks and risk factors for mother-child transmission of SARS-CoV-2 in utero, intra and peripartum, and the prevalence and persistence of the viral particles or immunological response in breast milk, amniotic fluid, cord blood, placenta, vaginal fluids and faeces in women and their babies (nasopharyngeal/throat swabs, blood, saliva, faeces).
- To assess the effects of interventions to prevent COVID-related complications.
- To determine if the rates of prevalence, clinical manifestations, outcomes and risk factors vary by: a) screening and testing strategy for SARS-CoV-2, b) selection of populations, c) risk status of the included women, timing of exposure (first, second, third trimester, postpartum), World Bank economic region (low, middle and high income) and quality of studies (low, high).

METHODS

Our systematic review protocol is registered in Prospero (CRD42020178076).[15] We will regularly repeat the searches, data extraction and analyses as described in Figure 1.

For peer review only - http://bmjopen.bmj.com/site/about/guidelines.xhtml

Literature search

We will carry out a systematic search on the World Health Organisations (WHO) Database of publications on COVID-19, the EPPI-Centre map of the current evidence on COVID-19, Cochrane databases, China National Knowledge Infrastructure (CNKI), Wanfang and preprint databases (ArXiv, BiorXiv, medRxiv, search.bioPreprint), the reference lists of included studies, relevant systematic reviews and guidelines published by national and international professional societies, COVID-19 research websites,[16,17] and follow blogs dedicated to the identification of primary case reports, case series, observational studies or randomised-controlled trials describing women affected by COVID-19 in pregnancy. We will also link with established groups conducting surveillance and research studies in pregnant women with COVID-19 to access their aggregate study data.[18-20] There will be no language restrictions. The search findings will be exported to Covidence (http://covidence.org/), an online programme which facilitates study selection and screening, recommended by the Cochrane Collaboration.[21] Our search will be updated weekly, and we shall review this frequency every two months. The search syntax for the Pubmed database is provided in Appendix 1

Study selection, quality assessment and data extraction

Study selection will be a two-stage process: titles and/or abstracts of studies will be screened first, followed by evaluation of full texts for eligibility by two independent reviewers. Disagreements will be resolved through discussion or by consulting a third reviewer. We will include all cohort studies (with at least 10 participants) and randomised trials reporting on clinical outcomes relating to SARS-CoV-2 infection involving pregnant and postpartum (which includes post-miscarriage/abortion period) women and their babies. We defined cohort studies as those that included women based on exposure, followed-up over specified

For peer review only - http://bmjopen.bmj.com/site/about/guidelines.xhtml

time and reported outcomes.[22] Case reports and case series will only be included to answer the research questions relating to mother-to-child transmission.

We will look for serological (IgM) and/or RT-PCR (reverse transcriptase polymerase chain reaction) confirmation of infection in amniotic fluid, placenta, cord, newborn and maternal blood, and newborn and maternal respiratory secretions (at birth, 24 and ≥48 hours after birth) to distinguish between congenital, intrapartum and postpartum transmission. Women with a respiratory sample (naso/oropharyngeal swab or tracheal/bronchoalveolar lavage) positive for SARS-CoV-2 RT-PCR or positive SARS-CoV-2 serological tests will be considered to have confirmed COVID-19. We will also assess risk factors such as mode of delivery, maternal disease severity, gestational age of maternal infection, preterm delivery, rooming-in and breast feeding on mother-to-child transmission. Women with a clinical diagnosis based on chest computerised tomography (CT) or radiograph or other features will be considered as suspected COVID-19. Table 1 provides the details of risk factors, maternal, and perinatal outcomes that will be evaluated in the review.

For peer review only

For peer review only - http://bmjopen.bmj.com/site/about/guidelines.xhtml

Table 1. Study participants, risk factors and outcomes evaluated in the living systematic review on COVID-19 in pregnant and postpartum women

**Population**	Pregnant/ postpartum/ postabortal women with suspected or confirmed COVID-19 infection
**Risk factors**	**Maternal**<br>Age, ethnicity, pre-existing medical conditions (including diabetes, chronic hypertension, asthma and COPD), smoking, immunosuppression, gestational diabetes, symptoms, hypertensive disorders in pregnancy (pre-eclampsia, pregnancy induced hypertension), BMI (Body Mass Index) ≥ 30, multiple pregnancy, in vitro fertilisation, parity, gestational age, mode of delivery, pregnancy status (pregnant or delivered), Reproductive tract infections, symptoms and abnormal lab results.
**Clinical manifestations**	**Symptoms and signs**<br>Cough, fever, breathlessness, sputum, myalgia, fatigue, diarrhoea, headache, sore throat, chest pain, rigor, ageusia, anosmia, nausea or vomiting, Sequential Organ Failure Assessment (SOFA), Quick SOFA (qSOFA), asymptomatic presentation<br>**Laboratory**<br>White cell count, lymphocyte count, haemoglobin, anaemia, platelet count, albumin, ALT, AST, C-reactive protein, creatinine, lactate dehydrogenase, creatinine kinase, high-sensitivity cardiac troponin, prothrombin time, D-dimer, serum, ferritin, interleukin-6, procalcitonin<br>**Radiological**<br>Consolidation, ground-glass opacity, bilateral pulmonary infiltration, unilateral pulmonary infiltration, abnormal chest X-Ray, abnormal chest CT
**Outcomes**	**Maternal COVID-related outcomes**<br>Mortality: all-cause mortality, COVID-specific mortality<br>Clinical Respiratory Syndrome: pneumonia, respiratory failure, ARDS (Acute Respiratory Distress Syndrome), severe pneumonia; invasive ventilation, non-invasive ventilation, oxygenation; long-term respiratory outcomes<br>Time from illness onset to outcome (death, recovery)<br>Hospitalisation: admission to ICU (Intensive Care Unit), admission to hospital, ICU length of stay<br>Organ Failure: sepsis, septic shock, cardiac failure, coagulopathy, thromboembolism, acute cardiac injury, acute kidney injury, acute hepatic failure, cytokine storm syndrome (haemophagocytic lymphohistiocytosis), hypoproteinaemia, acidosis, central nervous system manifestations, secondary infection, duration of viral shedding<br>Delirium, acute neuropsychiatric emergency, agitation, anxiety, depression, psychosis<br>**Pregnancy-related outcomes**<br>Preterm delivery (<37w, spontaneous preterm delivery, induced preterm birth), preterm-premature rupture of membranes, prelabour rupture of membranes at (or near) term, miscarriage (spontaneous), induced abortion, mode of delivery, induction of labour, chorioamnionitis, wound infection, pregnancy-induced hypertension, gestational diabetes, antepartum haemorrhage, postpartum haemorrhage<br>**Offspring outcomes**<br>Stillbirth, neonatal death (early, late), foetal distress, foetal growth restriction post infection, Apgar score at 1', 5'; cord blood pH, gestational age at delivery, birthweight, LGA (large-for-gestational age), SGA (small-for-gestational age), congenital malformation, HIE (hypoxic ischaemic encephalopathy), neonatal seizures, neonatal infection (other than COVID), neonatal sepsis, neonatal asphyxia, DIC (Disseminated Intravascular Coagulation), NEC (Necrotising Enterocolitis), RDS (Respiratory Distress Syndrome), admission to the neonatal unit, length of stay in neonatal unit<br>**Mother-to-child transmission outcomes**<br>Evidence of virus in amniotic fluid, cord blood, placenta, placental membranes, vaginal fluid, breast milk, neonatal throat swabs, maternal and neonatal faeces and saliva samples; IgM antibodies in cord blood, neonatal blood<br>Duration of viral shedding after COVID-19 symptoms onset and after clinical resolution of signs/symptoms in mother and in newborn

For peer review only

We will assess the risk of bias for included cohort studies using the Newcastle Ottawa Scale for comparative cohorts,[23] the Cochrane Risk of Bias 2 (RoB 2) tool for randomised trials,[24] QUADAS-2 for diagnostic accuracy studies,[25] and the tool outlined by Hoy et al. for prevalence studies.[26] A pre-piloted form will be used for data extraction of the included studies. Two reviewers will independently extract data and disagreements will be resolved by consensus or by consulting a third reviewer. We will assess for duplication of the data by comparing characteristics of the mother or baby and the settings of the studies. If required, we will contact the authors of primary studies for clarification about duplicate data. Where possible, we have planned a semi-automated process to identify duplicates and facilitate rapid update of search and data extraction.

Analysis

We will undertake narrative syntheses and perform aggregate meta-analyses when there are at least two studies with minimal clinical heterogeneity. Dichotomous outcomes will be summarised as proportions, odds ratios (OR) and continuous outcomes as standardised mean differences. We will use random effects model for the analysis when the number of studies permits to estimate between-study variance. To summarise proportions, we will use Freeman-Tukey transformation to stabilize variances while dealing with studies with zero events. We will provide 95% confidence intervals (CI) and predictive intervals (PI) to report on the precision of estimates and to aid the interpretation of heterogeneity. Heterogeneity will be reported as I^2 and tau-squared statistics.

Pre-planned subgroup analysis will be by a) suspected/probable or confirmed COVID-19, b) diagnosis in pregnancy or postnatal period, c) trimester of diagnosis (first, second or third), d)

For peer review only - http://bmjopen.bmj.com/site/about/guidelines.xhtml

country income-level (high or low- and middle-income country), e) screening strategies (universal, symptom-based or risk-based testing), f) maternal risk status (low or high) and g) study quality (low or high). We will undertake additional sensitivity analysis to explore the effects of different populations (unselected, selected) and by excluding women with suspected COVID-19 and studies at high risk of bias. We will use trial sequential analysis to control for type I and II errors while accounting for updating estimations of between-study heterogeneity. A priori assumptions on statistical power, minimal clinically relevant effect and heterogeneity between trials will be used to define maximum sample size to detect such an effect. Boundaries of statistical significance according to sample size will be defined and used to determine statistical significance for each systematic review update result.

We have established a pool of peer reviewers to rapidly assess the findings. The initial pre-peer reviewed findings will be published in a dedicated website, followed by the full findings when peer review is complete. We will simultaneously submit our work for publication in scientific journals with clear reference to the version of the living systematic review provided in that submission. Where journals allow, we will update our findings in the journals at set intervals required by the journal.

Patient and public involvement

Katie's Team Patients and Public Involvement group- a dedicated women's research and health advisory group, were involved in the design of the protocol, and will contribute to the interpretation and dissemination of the result.

DISCUSSION

For peer review only - http://bmjopen.bmj.com/site/about/guidelines.xhtml

Our living systematic review will address key research questions relevant to SARS-CoV-2 infection in pregnancy and postpartum period. Our review is based on a prospective protocol with plans to continuously update all review processes from search to publication at specific time points. The findings of the review will directly inform living guidelines and policies as new evidence emerge.

Our detailed literature search is a major strength of the review. In addition to the WHO COVID-19 database, our links with Cochrane Fertility and Gynaecology and EPPI Centre groups, and additional searches for studies mapped by dedicated websites as well as blogs and social media networks means that the chance of missing relevant studies are small. Through our networks with key collaborators in the WHO MNCAH (Maternal, neonatal, child and adolescent health) COVID-19 research group,[27] and relevant working groups, we will be able to access and include unpublished data. Our collaborative links with researchers in China provides access to Chinese language databases, so that these studies are not missed.

We have developed the protocol to ensure that all stages of the review are robust by adhering to recommended methods for conduct and reporting of systematic reviews.[28] By restricting case reports and series to the research question on mother-to-child transmission alone, we will not only ensure that relevant cases are not missed, but also avoid biased estimates for other research questions on risk factors and prevalence. Determination of mother-to-child SARS-CoV-2 transmission is particularly challenging since there is no consensus on what constitutes intrauterine, intrapartum, and postpartum transmission (including breast-milk versus horizontal transmission).[6] To maximise data collection addressing this question, we will include case reports and case series for this part of the review. We will collect data on all

For peer review only - http://bmjopen.bmj.com/site/about/guidelines.xhtml

types of samples to enable evaluation of varying definitions of the evidence required for confirmation of mother-to-child SARS-CoV-2 transmission and timing.

Our work is subject to some limitations. Our protocol has been developed based on our current knowledge of the COVID-19. As the pandemic unfolds, emerging new evidence may require changes in the protocol. The systematic review will be influenced by the characteristics of the individual studies, which may comprise heterogeneous populations, definitions of COVID-19, sampling frames, test strategies for diagnosis, definitions and reporting of outcomes. We have addressed these challenges by clearly defining the inclusion criteria, and by exploring heterogeneity through sensitivity and subgroup analyses. The findings will be reported as 95% CI to communicate the uncertainty around the pooled estimates and prediction intervals (PI) to anticipate the variability in new study estimates. Given the urgency of the situation in the pandemic era, many studies are published as preprints, often followed by a full publication at a later date. The living systematic review needs to be responsive to any changes in data between the preprint to full publication stage. Furthermore, there is a risk of duplicate data, as individual studies may report the findings of the same mother-baby data that have been published elsewhere. We plan to exclude studies with suspected duplicate data, and contact the authors of the primary studies for clarification. This review may be subject to publication bias, since studies with perceived positive results may be published faster than those with perceived negative results. We will continuously review registries of randomised controlled trials to detect trials that should have reported results but that have not done so and will contact corresponding authors to obtain results.

Many of the automated tools for living systematic review are mainly developed for reviews on randomised trials (RCT), and not for observational studies.[29] Unlike traditional systematic

For peer review only

reviews, living systematic reviews require substantial investment in time and human resources, and resources to regularly update the findings. Sustained funding is required to maintain the same level of output over a longer period of time. This is particularly challenging, as the publication rate of studies on SARS-Cov-2 infection in pregnancy and postpartum is likely to increase exponentially. To ensure that all relevant studies are identified in a timely manner, in addition to traditional searches, we will use our collaborations with other global efforts to map studies on COVID-19 and pregnancy and postpartum, and automated alerts to identify new evidence when it gets published. Given the wide scope of this review, numerous reviewers will be involved, requiring clear operating procedures and pathways in place for workflow, training, monitoring and quality assessment. The necessity to swiftly publish the collated evidence need to be balanced against publishing the findings after peer review. To be able to sustain the level of reviewer turn-around every two weeks will be a challenge.

To-date, apart from Cochrane, very few journals provide specific guidelines for publication of living systematic reviews and its subsequent update.[30] Various models of publication have been considered.[31] The first model is similar to the Cochrane reviews, where with each new update (usually done in yearly intervals), there is a new publication with a new version and DOI in PubMed with linked updates between versions. This model can also take into account any changes in authors between the versions.[32] However, in the current situation of rapidly evolving evidence, this is likely to result in numerous publications, even if reported on a monthly basis. In the second model, the introduction and methods of the main manuscript does not change, with only changes in the results section, which is written in such a way that most of the information is provided in Tables and Figures that are revised along with the abstract. The discussion section of the newer version can incorporate a paragraph on the

For peer review only - http://bmjopen.bmj.com/site/about/guidelines.xhtml

implications of new findings. The manuscript will be less resource intensive to prepare in the second model, but the original version should have been written in a generic manner to accommodate new information emerging in subsequent versions. In another model, the findings of subsequent updated analyses appear as new appendices, with no changes in the original abstract or manuscript. While this model requires less efforts from authors, editors and peer reviewers, it can mistakenly provide inaccurate old evidence if readers only access the abstract. Furthermore, this model makes it difficult to add or remove authors according to the changes in their contribution to subsequent versions.

In the midst of the current pandemic, much still remains unknown about how SARS-CoV-2 infection affects pregnant and postpartum women and their babies compared to reproductive aged non-pregnant women. It is essential that clinicians' decisions to manage pregnant and postpartum women and their babies with COVID-19 are guided by the evidence. Our living systematic review is well suited to rapidly provide updated findings for translation into clinical practice. The flexibility of the living systematic review needs to be matched by a willingness of journal editors and guideline makers to provide a framework that allows rapid dissemination of the new findings.

Ethics and dissemination

No ethical concerns. The findings will be disseminated through a designated website, publications, presentations in webinars and social media.

Data statement

Data sharing not applicable as no datasets generated and/or analysed for this study

Author contributions

All authors contributed to the development of the protocol and writing of the manuscript.

For peer review only - http://bmjopen.bmj.com/site/about/guidelines.xhtml

For peer review only

MY, LD, TK, SC - wrote the first draft of the manuscript

JA, ES - designed the study

DC, SL - reviewed the manuscript and were involved in quality assessment and data extraction. They have approved the final version of the manuscript.

ACL, AD, DZ, RB - reviewed the manuscript and are involved in study selection, and they have approved the final version of the manuscript.

XQ, MY, MvW, EK, EvL, LM, HK, AK, ST, JT, VB, NB, EK, CRK, AT, PRS, HPH, OTO - reviewed the manuscript, contributing critical changes, and they have approved the final version of the manuscript.

JZ - reviewed the manuscript and will conduct the statistical analysis. He approved the final version of the manuscript.

MB - designed the study, and reviewed the manuscript, and has approved the final version of the manuscript.

ST - conceived and designed the study, and reviewed the manuscript and approved the final version of the manuscript. ST is the guarantor.

All authors reviewed the manuscript, contributing critical changes, and approved the final version of the manuscript.

Funding statement

The work is partially funded by the World Health Organization. The authors alone are responsible for the views expressed in this manuscript and they do not necessarily represent the decisions, policy or views of the funding bodies. The views of the funding bodies have not influenced the content of this manuscript.

Competing interests

None

References

1. WHO announces COVID-19 outbreak a pandemic: World Health Organisation [Internet]; 2020. https://www.euro.who.int/en/health-topics/health-emergencies/coronavirus-covid-19/news/news/2020/3/who-announces-covid-19-outbreak-a-pandemic (accessed Jun 11 2020).
2. COVID-19 Dashboard: John Hopkins University [Internet]. https://coronavirus.jhu.edu/map.html (accessed Apr 21 2020).
3. Rajgor DD, Lee MH, Archuleta S, et al. The many estimates of the COVID-19 case fatality rate. *Lancet Infect Dis* 2020 doi: 10.1016/S1473-3099(20)30244-9
4. Mor G, Cardenas I. The immune system in pregnancy: a unique complexity. *Am J Reprod Immunol* 2010;63(6):425-33. doi: 10.1111/j.1600-0897.2010.00836.x

For peer review only - http://bmjopen.bmj.com/site/about/guidelines.xhtml
For peer review only

5. Schwartz DA, Graham AL. Potential Maternal and Infant Outcomes from (Wuhan) Coronavirus 2019-nCoV Infecting Pregnant Women: Lessons from SARS, MERS, and Other Human Coronavirus Infections. *Viruses* 2020;12(2) doi: 10.3390/v12020194
6. Blumberg DA, Underwood MA, Hedriana HL, et al. Vertical Transmission of SARS-CoV-2: What is the Optimal Definition? *Am J Perinatol* 2020 doi: 10.1055/s-0040-1712457
7. Covid-19 and pregnancy. *BMJ* 2020;369:m1672. doi: 10.1136/bmj.m1672
8. Kmietowicz Z. Covid-19: Half of pregnant women in UK hospitals are from ethnic minorities. *BMJ* 2020;369:m2266. doi: 10.1136/bmj.m2266
9. Millett GA, Jones AT, Benkeser D, et al. Assessing Differential Impacts of COVID-19 on Black Communities. *Ann Epidemiol* 2020 doi: 10.1016/j.annepidem.2020.05.003
10. Knight M, Bunch K, Vousden N, et al. Characteristics and outcomes of pregnant women admitted to hospital with confirmed SARS-CoV-2 infection in UK: national population based cohort study. *BMJ* 2020;369:m2107. doi: 10.1136/bmj.m2107
11. Haghani M, Bliemer MCJ, Goerlandt F, et al. The scientific literature on Coronaviruses, COVID-19 and its associated safety-related research dimensions: A scientometric analysis and scoping review. *Saf Sci* 2020:104806. doi: 10.1016/j.ssci.2020.104806
12. Lee AY, Lin MW. Rapid publishing in the era of coronavirus disease 2019 (COVID-19). *Med J Aust* 2020 doi: 10.5694/mja2.50617
13. Knottnerus JA, Tugwell P. Methodological challenges in studying the COVID-19 pandemic crisis. *J Clin Epidemiol* 2020;121:A5-A7. doi: 10.1016/j.jclinepi.2020.04.001
14. Shokraneh F, Adams CE. Study-based registers of randomized controlled trials: Starting a systematic review with data extraction or meta-analysis. *Bioimpacts* 2017;7(4):209-17. doi: 10.15171/bi.2017.25
15. Allotey J, Bonet M, Zamora J, et al. COVID-19 in pregnant women: a Living Systematic Review on prevalence, presentation, prognosis and treatment. PROSPERO 2020 CRD42020178076. *Prospero [Internet]* 2020
16. COVID-19, Maternal and Child Health, Nutrition: John Hopkins Center for Humanitarian health [Internet]. http://hopkinshumanitarianhealth.org/empower/advocacy/covid-19/covid-19-children-and-nutrition/ (accessed Jun 9 2020).
17. COVID-19 Research Community: ResearchGate [Internet]. https://www.researchgate.net/community/COVID-19 (accessed Jun 9 2020).
18. COVID-19: a living systematic map of the evidence: Eppi-centre [Internet]. http://eppi.ioe.ac.uk/cms/Projects/DepartmentofHealthandSocialCare/Publishedreviews/COVID-19Livingsystematicmapoftheevidence/tabid/3765/Default.aspx (accessed Jun 11 2020).
19. NIPH systematic and living map on COVID-19 evidnce: Norwegian Institute of Public Health [Internet]. https://www.nornesk.no/forskningskart/NIPH_mainMap.html (accessed Jun 11 2020).
20. Advanced Search: Cochrane Library. https://www.cochranelibrary.com/advanced-search (accessed Jun 11 2020).
21. Covidence: Cochrane. https://community.cochrane.org/help/tools-and-software/covidence (accessed Jun 14 2020).
22. Dekkers OM, Egger M, Altman DG, et al. Distinguishing case series from cohort studies. *Ann Intern Med* 2012;156(1 Pt 1):37-40. doi: 10.7326/0003-4819-156-1-201201030-00006
23. Wells G, Shea B, O'Connell D, et al. The Newcastle-Ottowa Scale (NOS) for assessing the quality of nonrandomised studies in meta-analysis: The Ottowa Hospital Research Institute; 2013. http://www.ohri.ca/programs/clinical_epidemiology/oxford.asp (accessed Jun 11 2020).
24. Sterne JAC, Savovic J, Page MJ, et al. RoB 2: a revised tool for assessing risk of bias in randomised trials. *BMJ* 2019;366:l4898. doi: 10.1136/bmj.l4898

For peer review only - http://bmjopen.bmj.com/site/about/guidelines.xhtml

25. Whiting PF, Rutjes AW, Westwood ME, et al. QUADAS-2: a revised tool for the quality assessment of diagnostic accuracy studies. *Ann Intern Med* 2011;155(8):529-36. doi: 10.7326/0003-4819-155-8-201110180-00009
26. Hoy D, Brooks P, Woolf A, et al. Assessing risk of bias in prevalence studies: modification of an existing tool and evidence of interrater agreement. *J Clin Epidemiol* 2012;65(9):934-9. doi: 10.1016/j.jclinepi.2011.11.014
27. Maternal, newborn, child and adolescent health (MNCAH) consolidated data portal: World Health Organisation [Internet]. https://www.who.int/maternal_child_adolescent/epidemiology/mncah-data-platform/en/ (accessed Jun 11 2020).
28. Moher D, Liberati A, Tetzlaff J, et al. Preferred reporting items for systematic reviews and meta-analyses: the PRISMA statement. *BMJ* 2009;339:b2535. doi: 10.1136/bmj.b2535
29. Khamis AM, Kahale LA, Pardo-Hernandez H, et al. Methods of conduct and reporting of living systematic reviews: a protocol for a living methodological survey. *F1000Res* 2019;8:221. doi: 10.12688/f1000research.18005.2
30. Brooker J, Synnot A, Mcdonald S, et al. Guidance for the production and publication of Cochrane living systematic reviews: Cochrane Reviews in living mode. 2019. https://community.cochrane.org/sites/default/files/uploads/inline-files/Transform/201912_LSR_Revised_Guidance.pdf.
31. Elliott JH, Synnot A, Turner T, et al. Living systematic review: 1. Introduction-the why, what, when, and how. *J Clin Epidemiol* 2017;91:23-30. doi: 10.1016/j.jclinepi.2017.08.010
32. Cumptsom M, Chandler J. Updating a review. In: Higgins JPT TJ, Chandler J, Cumpston M, Li T, Page MJ, Welch VA, ed. Cochrane Handbook for Systematic Reviews of Interventions version 60 (updated August 2019): Cochrane, 2019.

Figure Legend

Figure 1: Steps in the living systematic review (LSR) on COVID-19 in pregnant and postpartum women

For peer review only

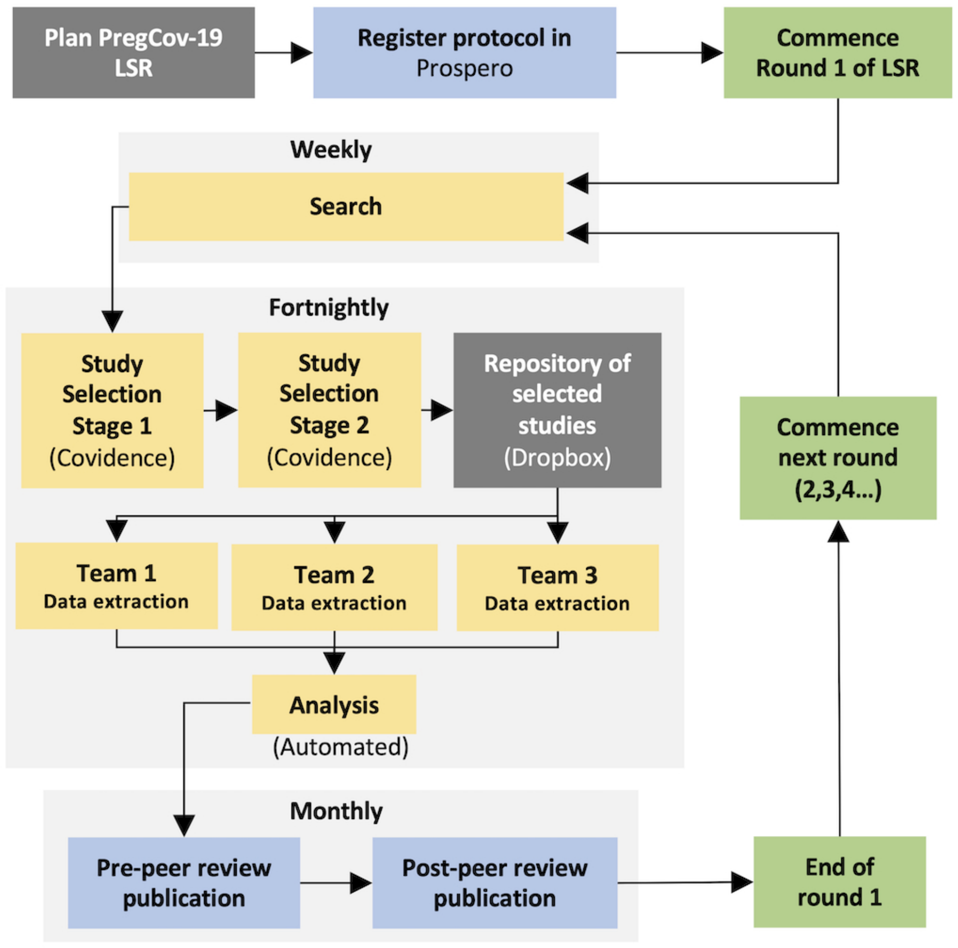

Fig 1. Steps in the living systematic review (LSR) on COVID-19 in pregnant and postpartum women

90x90mm (300 x 300 DPI)

Appendix 1: Pubmed search strategy to be used in the living systematic review on COVID-19 in pregnant and recently pregnant women

Item	Term
1	pregnancy/
2	pregnan*.tw.
3	neonatal.tw.
4	perinatal.tw.
5	mothers/.
6	mother.tw.
7	maternal.tw.
8	obstetric.tw.
9	infant, newborn/
10	infant.tw.
11	newborn.tw.
12	child*.tw.
13	or/1-12
14	COVID-19.tw.
15	COVID-2019.tw.
16	severe acute respiratory syndrome coronavirus 2.tw.
17	2019-nCoV.tw.
18	SARS-CoV-2.tw.
19	2019nCoV.tw
20	or/14-19
21	coronavirus.tw.
22	2019/12.pd
23	2020.pd.
24	or/22-23
25	21 and 24
24	or/20-25
25	13 and 24

For peer review only

PRISMA-P (Preferred Reporting Items for Systematic review and Meta-Analysis Protocols) 2015 checklist addressed in the living systematic review protocol on COVID-19 in pregnant and recently pregnant women

Section and topic	Item No	Checklist item	Page No.
**ADMINISTRATIVE INFORMATION**			
Title:			
Identification	1a	Identify the report as a protocol of a systematic review	1
Update	1b	If the protocol is for an update of a previous systematic review, identify as such	Not applicable
Registration	2	If registered, provide the name of the registry (such as PROSPERO) and registration number	4
Authors:			
Contact	3a	Provide name, institutional affiliation, e-mail address of all protocol authors; provide physical mailing address of corresponding author	1-2
Contributions	3b	Describe contributions of protocol authors and identify the guarantor of the review	16
Amendments	4	If the protocol represents an amendment of a previously completed or published protocol, identify as such and list changes; otherwise, state plan for documenting important protocol amendments	Not applicable
Support:			
Sources	5a	Indicate sources of financial or other support for the review	16
Sponsor	5b	Provide name for the review funder and/or sponsor	16
Role of sponsor or funder	5c	Describe roles of funder(s), sponsor(s), and/or institution(s), if any, in developing the protocol	16
**INTRODUCTION**			
Rationale	6	Describe the rationale for the review in the context of what is already known	6-7
Objectives	7	Provide an explicit statement of the question(s) the review will address with reference to participants, interventions, comparators, and outcomes (PICO)	7-8
**METHODS**			
Eligibility criteria	8	Specify the study characteristics (such as PICO, study design, setting, time frame) and report characteristics (such as years considered, language, publication status) to be used as criteria for eligibility for the review	9

For peer review only

Information sources	9	Describe all intended information sources (such as electronic databases, contact with study authors, trial registers or other grey literature sources) with planned dates of coverage	9
Search strategy	10	Present draft of search strategy to be used for at least one electronic database, including planned limits, such that it could be repeated	9
Study records:			
Data management	11a	Describe the mechanism(s) that will be used to manage records and data throughout the review	9
Selection process	11b	State the process that will be used for selecting studies (such as two independent reviewers) through each phase of the review (that is, screening, eligibility and inclusion in meta-analysis)	9
Data collection process	11c	Describe planned method of extracting data from reports (such as piloting forms, done independently, in duplicate), any processes for obtaining and confirming data from investigators	9
Data items	12	List and define all variables for which data will be sought (such as PICO items, funding sources), any pre-planned data assumptions and simplifications	10
Outcomes and prioritization	13	List and define all outcomes for which data will be sought, including prioritization of main and additional outcomes, with rationale	10
Risk of bias in individual studies	14	Describe anticipated methods for assessing risk of bias of individual studies, including whether this will be done at the outcome or study level, or both; state how this information will be used in data synthesis	10
Data synthesis	15a	Describe criteria under which study data will be quantitatively synthesised	11
	15b	If data are appropriate for quantitative synthesis, describe planned summary measures, methods of handling data and methods of combining data from studies, including any planned exploration of consistency (such as I^2, Kendall's τ)	11
	15c	Describe any proposed additional analyses (such as sensitivity or subgroup analyses, meta-regression)	11
	15d	If quantitative synthesis is not appropriate, describe the type of summary planned	11
Meta-bias(es)	16	Specify any planned assessment of meta-bias(es) (such as publication bias across studies, selective reporting within studies)	11
Confidence in cumulative evidence	17	Describe how the strength of the body of evidence will be assessed (such as GRADE)	11

For peer review only
